# Enhanced Majorana stability in a three-site Kitaev chain

**DOI:** 10.1038/s41565-025-01894-4

**Published:** 2025-03-31

**Authors:** Alberto Bordin, Chun-Xiao Liu, Tom Dvir, Francesco Zatelli, Sebastiaan L. D. ten Haaf, David van Driel, Guanzhong Wang, Nick van Loo, Yining Zhang, Jan Cornelis Wolff, Thomas Van Caekenberghe, Ghada Badawy, Sasa Gazibegovic, Erik P. A. M. Bakkers, Michael Wimmer, Leo P. Kouwenhoven, Grzegorz P. Mazur

**Affiliations:** 1 QuTech and Kavli Institute of NanoScience, Delft University of Technology, Delft, The Netherlands; 2 Quantum Machines, Tel Aviv-Yafo, Israel; 3 Department of Applied Physics, Eindhoven University of Technology, Eindhoven, The Netherlands; 4 Department of Materials, University of Oxford, Oxford, UK

**Keywords:** Superconducting devices, Superconducting properties and materials, Quantum information

## Abstract

Majorana zero modes are non-Abelian quasiparticles predicted to emerge at the edges of topological superconductors. A one-dimensional topological superconductor can be realized with the Kitaev model—a chain of spinless fermions coupled via *p*-wave superconductivity and electron hopping—which becomes topological in the long-chain limit. Here we realize a three-site Kitaev chain using semiconducting quantum dots coupled by superconducting segments in a hybrid InSb/Al nanowire. We investigate the robustness of Majorana zero modes under varying coupling strengths and electrochemical potentials, comparing two- and three-site chains realized within the same device. We observe that extending the chain to three sites enhances the stability of the zero-energy modes, especially against variations in the coupling strengths. This experiment lacks superconducting phase control, yet numerical conductance simulations with phase averaging align well with our observations. Our results demonstrate the scalability of quantum-dot-based Kitaev chains and its benefits for Majorana stability.

## Abstract (web summary)

A three-site Kitaev chain, constructed from three semiconducting quantum dots coupled by superconducting segments in a hybrid InSb/Al nanowire, shows enhanced robustness of edge zero-energy modes against variations in the coupling strengths or electrochemical potentials compared with a chain containing only two quantum dots.

## Main

The pursuit of topological superconductivity is motivated by its potential to enable decoherence-free quantum computing and high-fidelity quantum gates^1,2^. Topological superconductors host zero-energy subgap states known as Majorana zero modes (MZMs), which are predicted to exhibit non-Abelian exchange statistics. Unlike bosons or fermions, exchanging (braiding) MZMs alters their collective wavefunction in a manner that depends on the order of the exchanges, a key feature for topological quantum computation. However, naturally occurring topological superconductors are rare, making the ability to engineer such systems highly appealing. Over the past decade, numerous experimental platforms have emerged as potential realizations of topological superconductivity. These include proximitized Rashba nanowires^3–6^, chains of magnetic atoms on superconductors^7^, two-dimensional van der Waals heterostructures^8^, phase-biased Josephson junctions^9–11^ and iron-based superconductors^12^, among others^13,14^. Recently, a promising alternative approach has emerged, utilizing two quantum dots (QDs) coupled through a superconductor to form a minimal Kitaev chain^15–18^. Even the shortest, two-site Kitaev chain hosts a pair of MZMs^19^. While MZMs in long chains are expected to be resilient against local noise and chemical potential variations, MZMs in two-site Kitaev chains are different. Owing to their vulnerability against interdot coupling variations, they are referred to as poor man’s MZMs. In this work, we experimentally realize a three-site Kitaev chain in an array of three QDs coupled by two short InSb/Al hybrids. By setting one QD on- or off-resonance, we can switch from a three-site to a two-site chain configuration and compare the robustness of the emerging MZMs. Specifically, the three-site chain shows resilience to perturbations in coupling amplitudes and increased stability against variations in chemical potential. These findings are well captured by our numerical simulations. This result highlights the potential to scale these systems to longer chains, laying the groundwork for realizing topological superconductivity in superconductor–QD chains.

## Coupling QDs

**Fig. 1:**
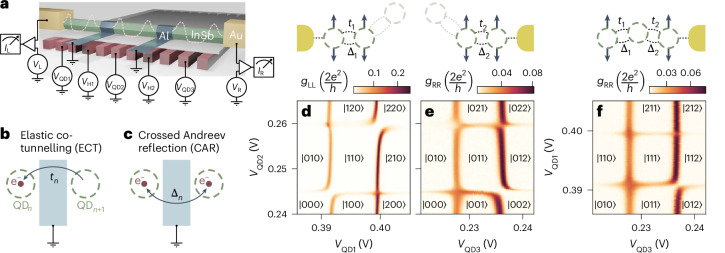
Strong *t*_*n*_ and Δ_*n*_ couplings between all the three QDs of the device. **a**, Illustration of the device. A semiconducting InSb nanowire (green) is placed on an array of eleven gates (red) and contacted by two Al (blue) and two Cr/Au (yellow) leads. The gates, separated from each other and from the nanowire by a thin dielectric, form a potential landscape defining three QDs, controlled by the plunger gate voltages *V*_QD1_, *V*_QD2_ and *V*_QD3_. The QDs are connected by two hybrid semiconducting–superconducting sections controlled by *V*_H1_ and *V*_H2_. The superconductors are separately grounded through room-temperature electronics, while the left and right normal probes are connected to corresponding voltage sources (*V*_L_, *V*_R_) and current meters (*I*_L_, *I*_R_). Differential conductances $({g}_{{\rm{L}}}\equiv \frac{{\mathrm{d}}{I}_{{\rm{L}}}}{{\mathrm{d}}{V}_{{\rm{L}}}},\,{g}_{{\rm{R}}}\equiv \frac{{\mathrm{d}}{I}_{{\rm{R}}}}{{\mathrm{d}}{V}_{{\rm{R}}}})$ are measured with standard lockin techniques. A scanning electron micrograph of the device is shown in Extended Data Fig. 5a. **b**,**c**, Schematic illustrations of ECT (**b**) (electron tunnelling between neighbouring QDs) and CAR (**c**) (creation or annihilation of two electrons into neighbouring QDs). **d**–**f**, QD–QD charge stability diagrams (where $| {n}_{1},{n}_{2},{n}_{3}\left.\right\rangle$ indicate the effective charge occupations). Zero-bias conductance is measured across two charge degeneracy points for every pair of QDs. Avoided crossings indicate strong coupling between each pair, while crossings signal that couplings between the dots are equalized^17^. **d** reports the QD_1_–QD_2_ charge stability diagram with QD_3_ kept off-resonance, **e** reports the QD_2_–QD_3_ charge stability diagram with QD_1_ kept off-resonance, while **f** reports the QD_1_–QD_3_ charge stability diagram with QD_2_ set close to resonance, as the schematics above indicate. In such schematics, whenever the *t*_*n*_ and Δ_*n*_ couplings are–in general not equalized, they are represented by two arcs. Off-resonance QDs are faint and displaced.

**Extended Data Fig. 1:**
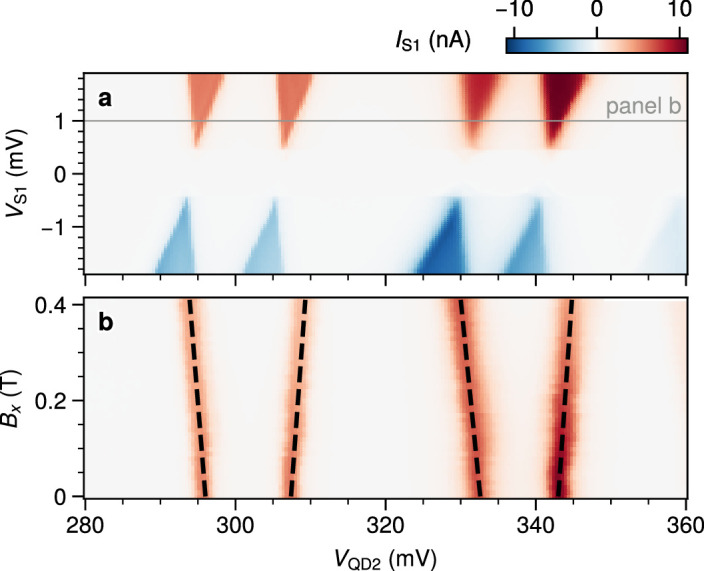
QD spin polarization. From the QD_2_ Coulomb diamonds shown in panel **a**, spanning two orbitals, we extract respectively charging energies of 3.3 and 2.5 meV and lever arms of 0.28 and 0.23. Using such lever arms, we can fit the *g*-factor from the *B*_*x*_ dependence at *V*_S1_ = 1 mV shown in panel **b**. The black dashed lines yield *g* = 45 ± 7, on par with our previous measurements^24^. With magnetic field *B* = *B*_*x*_ = 200 mT, this gives a Zeeman splitting *E*_Z_ = *gμ*_B_*B* = 0.5 meV, which is much bigger than our temperature broadening (few µeV) and our interdot couplings *t*_*n*_ and *Δ*_*n*_. If the QDs are strongly hybridized with the ABSs, then the *g*-factor is renormalized to lower values. Then, a lower bound to the QD *g*-factor is set by the ABS one, which is ~ 20, as estimated below in Extended Data Fig. 2 and in ref. ^17^ (a direct *g*-factor measurement of strongly hybridized QDs is reported in Ref. ^30^). This gives a lower bound *E*_Z_ > 0.2 meV for all our QDs. We note that the Zeeman splitting might vary from dot to dot, but as long as *E*_Z_ ≫ *k*_*B*_*T*, *t*_*n*_, *Δ*_*n*_, the QDs are well polarized. Finally, two further independent checks are consistent with a high Zeeman energy: first, the QD spectra shown in Extended Data Fig. 4 show isolated lines for all the QD resonances used for the experiment, secondly, the poor man’s Majorana spectra measured when detuning individual QDs, reported in the linked repository, do not show the extra features predicted for low Zeeman energy^18,55^.

**Extended Data Fig. 2:**
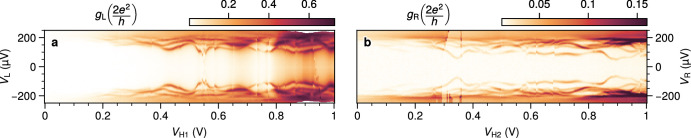
ABS spectroscopy. **a**. Spectroscopy of the left hybrid. **b**. Spectroscopy of the right hybrid. Both panels are measured at a fixed external magnetic field roughly parallel to the nanowire *B*_x_ = 200 mT and exhibit ABSs populating the spectrum. We chose a magnetic field intensity that is large enough to polarize the dots (≳ 100 mT) but small enough for the ABSs not to close the gap (≲ 300 mT). From the ABS energy, we estimate the ABS *g*-factor to be ~ 20. Both measurements are corrected for a dilution refrigerator line resistance of 7 k*Ω*.

To engineer a three-site Kitaev chain Hamiltonian^15,16^

1 $$H=\mathop{\sum }\limits_{n=1}^{3}{\mu }_{n}{c}_{n}^{\dagger }{c}_{n}+\mathop{\sum }\limits_{n=1}^{2}\left({t}_{n}{c}_{n}^{\dagger }{c}_{n+1}+{\Delta }_{n}{c}_{n}^{\dagger }{c}_{n+1}^{\dagger }+h.c.\right),$$

where ${c}_{n}^{\dagger }$ and *c*_*n*_ are the fermionic creation and annihilation operators, we need control over the on-site energies *μ*_*n*_, the hopping terms *t*_*n*_ and the pairing terms Δ_*n*_. In our semiconducting nanowire device, shown in Fig. 1a, three QDs are defined by an array of bottom gates, with *V*_QD1_, *V*_QD2_ and *V*_QD3_ controlling the electrochemical potentials *μ*_*n*_ of every QD. The hopping term *t*_*n*_ is realized by elastic co-tunnelling between the dots, whereas Δ_*n*_ is achieved through crossed Andreev reflection^16^, which splits Cooper pairs into two adjacent QDs^20–23^. Schematics of these two processes are depicted in Fig. 1b,c. To lift the spin degeneracy, as prescribed by the Hamiltonian of equation (1), we apply a magnetic field parallel to the nanowire axis (*B*_*x*_ = 200 mT). This leads to spin polarization of the QDs (Extended Data Fig. 1). The spin–orbit coupling in InSb nanowires induces spin precession, allowing for simultaneous occurrence of elastic co-tunnelling and crossed Andreev reflection across all spin configurations of the QDs^24,25^. Tunnelling spectroscopy of our semiconductor–superconductor hybrid segments (referred to as hybrids further in the text) is also performed and shown in Extended Data Fig. 2.

**Extended Data Fig. 3:**
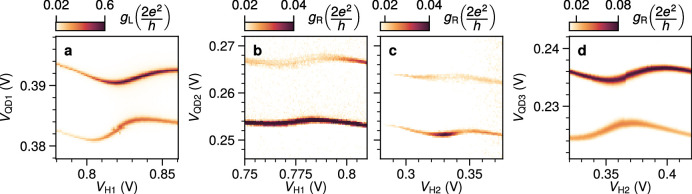
QD-ABS charge stability diagrams. **a**. Left zero-bias conductance as a function of *V*_QD1_ and *V*_H1_. **b, c, d**. Right zero-bias conductance as a function of *V*_QD2_ and *V*_H1_ (panel c), *V*_QD2_ and *V*_H2_ (panel c), *V*_QD3_ and *V*_H2_ (panel d). All panels show how a pair of QD resonances is modulated by the neigbouring hybrid gates, indicating QD-ABS hybridization^30^. Panels b and c are measured before forming a QD on the right; here there is a single tunneling barrier separating the right normal lead and the right hybrid so that it is possible to perform spectroscopy of QD_2_ from the right normal lead as long as the right bias *V*_R_ is smaller than the superconducting gap^49,50^.

In our previous work^26^, we confirmed the presence of *t*_1,2_ and Δ_1,2_ by detecting elastic co-tunnelling and crossed Andreev reflection across two hybrid segments with weakly coupled QDs. Here we target strong couplings: *t*_*n*_, Δ_*n*_ ≫ *k*_B_*T*, where *k*_B_ is the Boltzmann constant and *T* is the temperature. Indeed, the minimum value among *t*_*n*_ and Δ_*n*_ determines the amplitude of the topological gap in a long Kitaev chain^16,27^. To couple the QDs, we rely on the Andreev bound states (ABSs) populating the hybrids^28–30^. Measuring the zero-bias conductance on the left and the right of the device $({g}_{{\rm{L}}}\equiv \frac{{\mathrm{d}}{I}_{{\rm{L}}}}{{\mathrm{d}}{V}_{{\rm{L}}}},\,{g}_{{\rm{R}}}\equiv \frac{{\mathrm{d}}{I}_{{\rm{R}}}}{{\mathrm{d}}{V}_{{\rm{R}}}})$, we optimize the coupling site by site, as described in Methods and in Extended Data Fig. 3, until we see the appearance of several avoided crossings in the charge stability diagrams of Fig. 1d–f. Panels d and e show avoided crossings between QD_1_ and QD_2_ and between QD_2_ and QD_3_, respectively. Remarkably, the coupling between neighbouring QDs is strong enough to mediate interaction even between the outer QDs (panel f). We note that the coupling between QD_1_ and QD_3_ is mediated by the middle QD as it is suppressed if QD_2_ is moved off-resonance (see Supplementary Fig. 1 in the Supplementary Information).

## Tuning two-site Kitaev chains

After demonstrating strong coupling between the QDs, the next goal is to demonstrate the tunability of the chain. Ideally, elastic co-tunnelling and crossed Andreev reflection amplitudes should be balanced pairwise, setting

2 $$\left\{\begin{array}{rcl}{t}_{1}&=&{\Delta }_{1}\\ {t}_{2}&=&{\Delta }_{2}\end{array}\right.$$

**Fig. 2:**
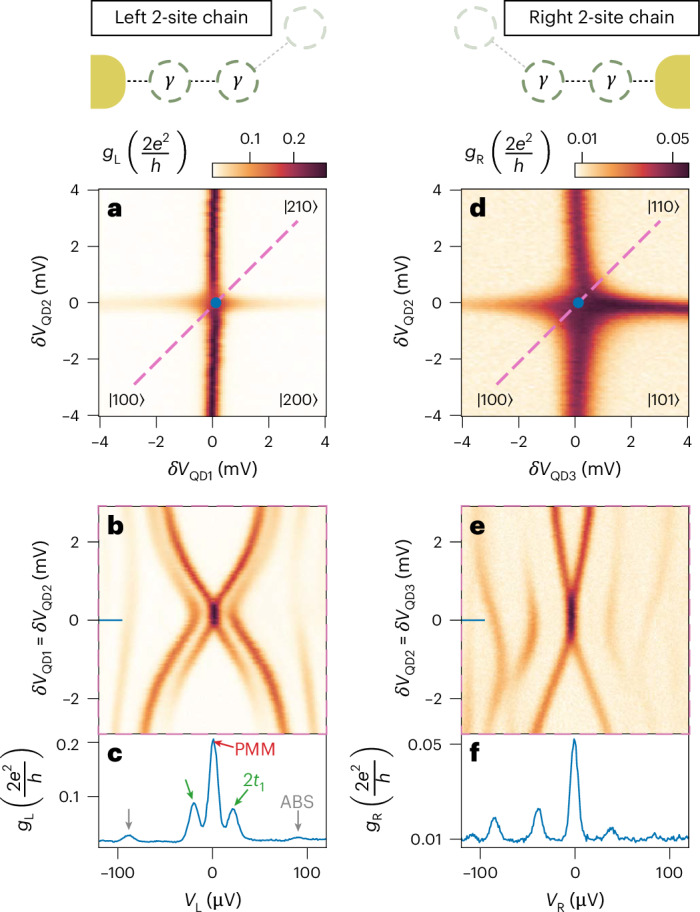
Two-site Kitaev chains tuned on both ends of the device. In the left column, QD_1_ and QD_2_ are on-resonance, while QD_3_ is being kept off-resonance as depicted in the schematic (*δV*_QD3_ = −5 mV). With *δV*_QD1/2/3_, we indicate the deviations from the crossing points, here happening at *V*_QD1_ = 0.3995 V, *V*_QD2_ = 0.2445 V and *V*_QD3_ = 0.2275 V. **a**, QD_1_–QD_2_ charge stability diagram at a sweet spot where *t*_1_ = Δ_1_. **b**, Conductance spectroscopy as a function of simultaneous detuning of QD_1_ and QD_2_. **c**, Line-cut depicting spectrum at *δV*_QD1_ = *δV*_QD2_ = 0 V illustrating a zero-bias peak, signature of a poor man's Majorana (red arrow) and a gap of ~20 μeV (green arrows). An ABS is visible at higher bias (grey arrows). Right column: QD_2_ and QD_3_ are kept on-resonance, while QD_1_ is kept off-resonance as depicted in the schematic (*δV*_QD1_ = −4 mV). **d**, QD_2_–QD_3_ charge stability diagram at a *t*_2_ = Δ_2_ sweet spot. **e**, Conductance spectroscopy as a function of simultaneous detuning of QD_2_ and QD_3_. **f**, Line-cut depicting spectrum at *δV*_QD2_ = *δV*_QD3_ = 0 V, illustrating a zero-bias peak and a gap of ~40 μeV.

We begin by illustrating in Fig. 2 how each condition of equation (2) can be individually met, with the constraint of keeping constant voltages on the three central gates forming QD_2_.

**Extended Data Fig. 4:**
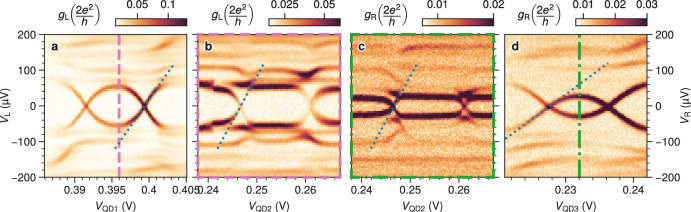
QD Spectroscopy. **a**. QD_1_ spectroscopy. The QD state appears as an eye-shape, while the ABSs of the left hybrid are visible at higher energies^30^. **b**. QD_2_ spectroscopy taken from the left probe. Here QD_1_ is kept in the middle of the pair of charge degeneracy points shown in panel a: *V*_QD1_ = 0.396 V. QD_1_ states appear as persistent lines at ≈ ± 60 *μ*V and mix with the ABS and QD_2_ spectra. **c**. QD_2_ spectroscopy taken from the right probe. Here *V*_QD3_ = 0.232 V. QD_3_ states appear as persistent lines at ≈ ± 25 *μ*V and mix with the ABS and QD_2_ spectra. Both panels show zero energy crossings at ≈ 0.246 and ≈ 0.2615 V, which we attribute to QD_2_ charge transitions. **d**. QD_3_ spectroscopy. We note that the eye shape is smaller compared to QD_1_, which implies a lower lever arm. The lever arms *α*_*n*_ are extracted for all QDs from the slopes of the fitted blue dotted lines; here we find *α*_1_ = 0.03, *α*_2_ = 0.025 and *α*_3_ = 0.014.

In the measurements of the left column of Fig. 2, QD_3_ is kept off-resonance, such that the low-energy behaviour of the chain is effectively two sites. When *t*_1_ = Δ_1_, we observe level crossing instead of repulsion in Fig. 2a (ref. ^17^) (see tuning procedures in Methods). The spectrum at the centre, shown in Fig. 2c, shows a zero-bias conductance peak corresponding to a poor man’s Majorana mode^19^, with the excitation gap being 2*t*_1_ = 2Δ_1_ ≈ 20 μeV. As pointed out in ref. ^19^, if *μ*_1_ and *μ*_2_ are detuned from 0, then the poor man’s Majoranas split quadratically from zero energy, as shown in Fig. 2b. Similarly, the right column of Fig. 2 studies the case where QD_1_ is kept off-resonance and the poor man’s Majorana pair appears on the right side of the device when *t*_2_ = Δ_2_. We note that the gap is ≈ 40 μeV, twice the left one. This is achieved with a higher degree of hybridization between the ABSs of the right hybrid and the neighbouring QDs (see Extended Data Fig. 4), resulting in higher coupling strengths as well as lower QD lever arms^30^. Although it is possible to tune the amplitudes of *t*_*n*_ and Δ_*n*_ to be all equal, we choose to focus on the scenario where they are equal only pairwise.

This approach allows us to identify spectral features arising from different coupling values in the chain.

## The three-site chain

**Fig. 3:**
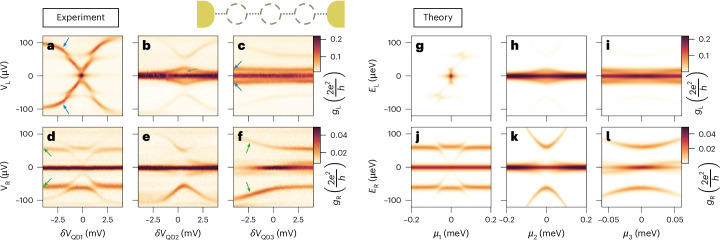
A three-site Kitaev chain. **a**–**c**, Conductance spectroscopy from the left lead, as a function of the detuning $\delta {V}_{{{\rm{QD}}}_{n}}$ of quantum dots QD_1_ (**a**), QD_2_ (**b**) and QD_3_ (**c**). By looking at the excited states when QD_3_ is off-resonance, we can estimate the left couplings to be 2*t*_1_ = 2Δ_1_ ≈ 20 μeV (blue arrows in **c**). **d**–**f**, Spectroscopy analogous to **a**–**c**, respectively, but measured from the right lead. When QD_1_ is off-resonance, we can estimate the left couplings to be 2*t*_2_ = 2Δ_2_ ≈ 60 μeV (green arrows in **d**). **g**–**l**, Numerical simulations corresponding to **a**–**f**, respectively, calculated by averaging differential conductances over uniformly distributed phase differences between the superconducting leads.

Having satisfied the pairwise condition of equation (2), we tune into the three-site Kitaev chain regime by setting all QDs on-resonance. Figure 3 shows the spectrum of such a system, tunnel-probed from the left and the right (first and second row, respectively), as a function of the detuning of every QD (first, second and third column).

The first observation is zero-bias conductance peaks manifesting on both ends of the device, remaining stable against the detuning of any constituent QD. Furthermore, spectroscopies from the left and the right reveal identical gate dispersions of the excited states, albeit with different intensities. Excited states originating from the left two sites are expected to couple more strongly to the left lead, while excited states originating from the right pair are expected to couple more strongly to the right one. Indeed, we identify excited states corresponding to 2*t*_1_ = 2Δ_1_ ≈ 20 μeV, marked by blue arrows in Fig. 3a,c. Such states disperse only as a function of *V*_QD1_ and *V*_QD2_ and have higher *g*_L_, signalling a higher local density of states. For the right side of the device, similar reasoning applies to the states marked by green arrows in Fig. 3d,f, from which we estimate 2*t*_2_ = 2Δ_2_ ≈ 60 μeV.

Importantly, we observe a finite conductance between the first excited state and the zero-bias peak (grey arrow in Fig. 3b). While we have successfully equalized the amplitudes of the coupling parameters, another significant parameter to consider is the phase difference between them. In the Kitaev chain Hamiltonian (equation (1)), the terms *t*_*n*_ and Δ_*n*_ are complex numbers, each with a distinct, non-trivial phase: ${t}_{n}=| {t}_{n}| {e}^{i{\phi }_{n,t}}$ and ${\Delta }_{n}=| {\Delta }_{n}| {e}^{i{\phi }_{n,\Delta }}$.

**Extended Data Fig. 5:**
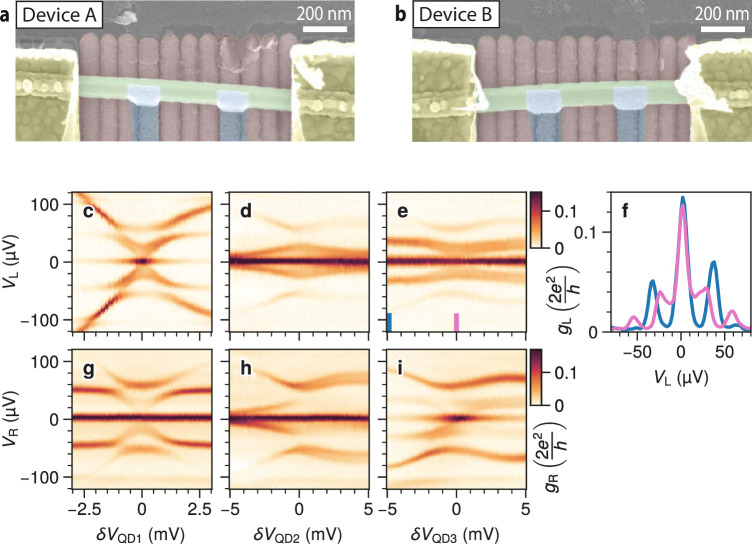
Second Device. **a**, **b**. False-colored scanning electron micrographs of the two devices. InSb nanowires (green) are deposited on top of an array of bottom gates (pink) and contacted by superconductors (blue) and normal metals (yellow). The nanofabrication details are reported in ref.^26^. **c-e, g-i**. Left and right tunneling spectroscopy of a device B tuned to the double sweet-spot condition of Eq. (2). Here, 2*t*_1_ = 2*Δ*_1_ ≈ 30 *μ* eV and 2*t*_2_ = 2*Δ*_2_ ≈ 60 *μ* eV. Such coupling strengths are tuned on purpose to values similar to the ones measured for the main text device (A). The remarkable similarity between this figure and panels a-f of Fig. 3 evidences the determinism and reproducibility of our tuning procedure across multiple devices. This device’s QDs, elastic co-tunneling and crossed Andreev reflection are characterized at zero external magnetic field in ref.^26^. **f**. Linecuts of panel c at *δV*_QD3_ = − 5 mV (blue) and 0 mV (pink) showing that when all QDs are on resonance there is no gap in the conductance (pink line) due to fast phase precession.

In the context of a two-site Kitaev chain, the consideration of these phases is redundant as they can be absorbed into the QD modes via a gauge transformation^16^. The scenario changes however with a three-site Kitaev chain, where only three out of the four phases can be similarly absorbed, leaving one phase as an independent parameter. In our system, the phase difference originates from the superconducting leads, which then translates into the phase difference between Δ_1_ and Δ_2_, as explained in Section B of the Supplementary Information. To understand the spectroscopic results presented in Fig. 3, we offer the following interpretation. Conceptually, the device’s central part is a Josephson junction, which does not exhibit any measurable supercurrent when the device is tuned in a three-site chain configuration (Supplementary Fig. 2). As a result, the junction behaves ohmically and can support an infinitesimal voltage difference. According to the second Josephson relation^31^, finite voltage bias in Josephson junctions induces phase precession: $\frac{{\mathrm{d}}\phi }{{\mathrm{d}}t}=\frac{2eV}{\hslash }$. In our experiment, the voltage bias between the two superconducting leads cannot be set to zero with arbitrary precision, owing to voltage divider effect, thermal fluctuations, finite equipment resolution and noise levels. We estimate the voltage difference to be on the order of *δV* ≈ 1 μV (Methods). The corresponding phase difference precesses with periods of ${T}_{\phi }=\frac{h}{2e\delta V} \approx 2$ ns. This is a very small timescale relative to the d.c. measurement time (~1 s). We thus assume that the spectra obtained for a three-site chain are uniformly averaged over possible phase differences. Figure 3g–l shows the average simulated conductance of 50 phase selections uniformly distributed from 0 to 2*π*. To calculate the differential conductances, we extend the system described by equation (1) to include couplings to external normal leads. We then apply the scattering matrix method to this extended system (see Methods for more details on this calculation). Within our interpretation, the zero-bias conductance peaks in the vicinity of the sweet spot (*μ*_*n*_ = 0, *t*_*n*_ = Δ_*n*_) are still induced by the three-site chain MZMs even in the presence of an uncertainty in phase *ϕ* (see Sections B and C of the Supplementary Information for a detailed analysis). Our theoretical model reproduces the features observed in the experiment accurately, despite having only a few parameters. As opposed to a spinful model treating the ABSs in the hybrids explicitly^32–34^, the effective spinless model that we are considering here only requires the fitting of the coupling to the leads *Γ*_L/R_; all other model parameters are estimated from independent measurements (Methods). We note that these observations have been replicated also on another nanowire device with similar values of *t*_*n*_ and Δ_*n*_, as presented in Extended Data Fig. 5.

## Enhanced stability

**Fig. 4:**
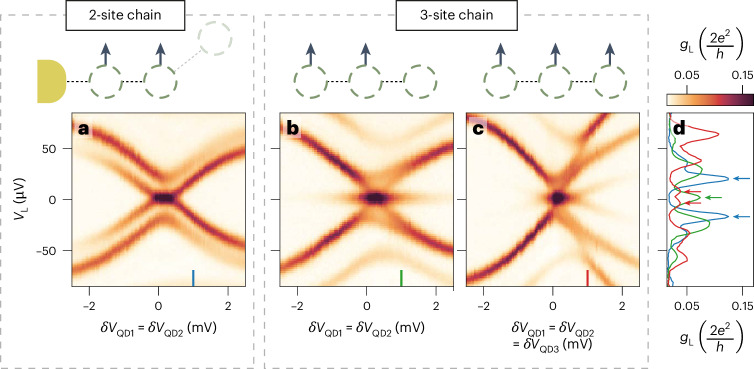
Stability of zero-energy states against electrochemical potential variations. Left conductance spectroscopy of the device in a two-site chain configuration (**a**) and three-site chain configuration (**b** and **c**). Theory simulations are shown in Extended Data Fig. 6. **a**, Spectrum of a two-site chain at the left of the device (as Fig. 2c) showing the splitting of poor man’s Majorana modes as a function of simultaneous detuning of QD_1_ and QD_2_. QD_3_ is off-resonance at *δV*_QD3_ = −5 mV. **b**, A three-site chain configuration where *δV*_QD3_ = 0 V. The zero-bias conductance peak persists over the full scanned range. See Extended Data Fig. 8 for similar measurements as a function of the detuning of any pair of QDs. Black arrows indicate which QDs are detuned simultaneously. **c**, Three-site chain spectrum as a function of simultaneous detuning *δV* of QD_1,2,3_. A visible splitting is observed once all the dots are detuned by *δV* = 1 mV. **d**, Line-cuts of previous panels taken at *δV* = 1 mV for a two-site chain (blue) and a three-site one (green and red). The arrows highlight the splitting of the zero-bias peak.

**Extended Data Fig. 6:**
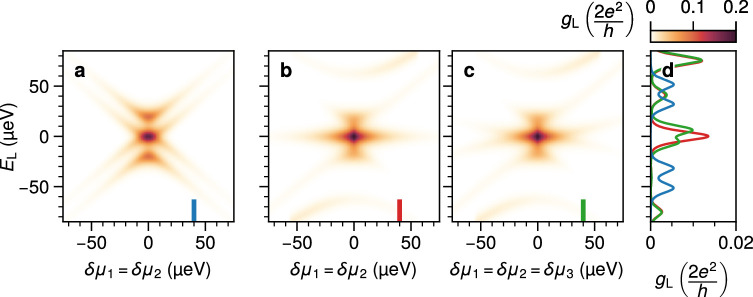
Simulation corresponding to Fig. 4. a-d. Theoretical simulation of Fig. 4 data with the spinless model of Eq. (1). All panels report the average conductance of 50 simulations with different phases on *Δ*_2_. The phase choices are uniformly distributed between 0 and 2*π*. Panel d displays linecuts at *μ* = 40 *μ* eV.

**Extended Data Fig. 7:**
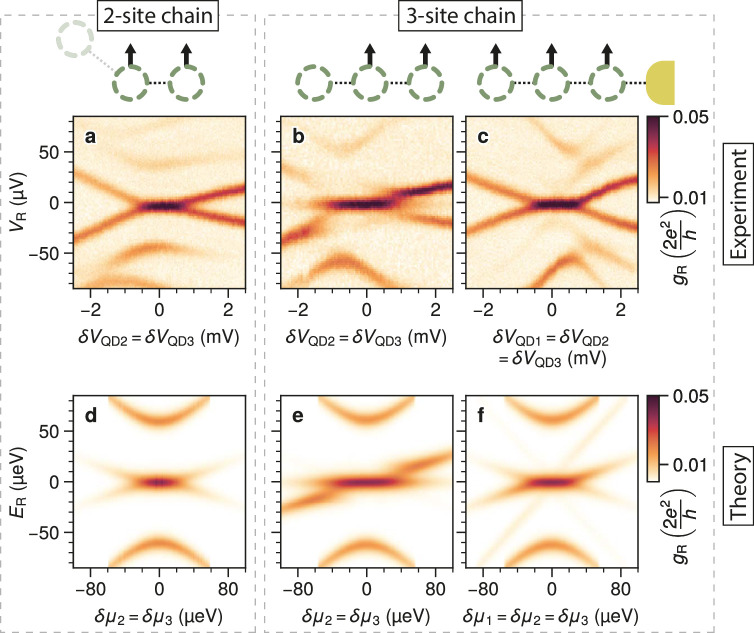
Right zero-bias peak stability against chemical potential variations. **a-c**. Tunneling spectroscopy measured from the right lead. **a**. Spectroscopy of a two-site Kitaev chain located on the right side of the device as a function of simultaneous detuning of QD_2_ and QD_3_. QD_1_ is off-resonance. **b**. When the leftmost dot is brought on-resonance, a three-site Kitaev chain is formed and the zero-bias conductance peak persists as QD_2_ and QD_3_ are detuned. **c**. Spectroscopy of the three-site chain against simultaneous detuning of all constituent quantum dots. **d-f**. Theoretical simulations of each detuning scenario with phase averaging. It shows good consistency with the experimental measurements.

**Extended Data Fig. 8:**
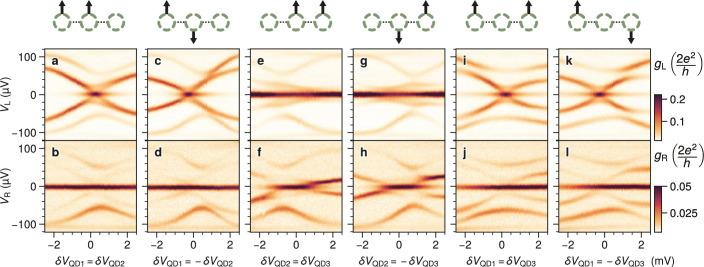
Zero bias peak persistence of a three-site chain while detuning any pair of QDs. Tunnelling spectroscopy from the left probe (top row) and the right one (bottom row). **a, b**. Symmetric detuning of QD_1_ and QD_2_. **c, d**. Anti-symmetric detuning of QD_1_ and QD_2_. **e-l**. Symmetric and anti-symmetric detuning of any other pair of QDs. Black arrows indicate which QDs are detuned together, and whether they are detuned in the same or opposite direction. All panels show a persistent zero-bias conductance peak over the full detuning range.

Figure 4 compares the robustness of two- and three-site chains against electrochemical potential variations. As shown in Fig. 2, detuning both QDs of two-site chains leads to the splitting of the poor man’s Majorana modes. In panels a and b of Fig. 4, we compare such a scenario with the detuning of the same two QDs in a three-site chain. Apart from *V*_QD3_, all the gate settings are identical, but the spectrum measured from the left probe shows for the three-site chain a stable zero-bias peak. To split the zero-energy modes of three-site chains, all QDs need to be detuned, as shown in panel c, and even in this case they disperse slower compared with the two-site scenario (as seen in panel d). As we demonstrate in the Supplementary Information (Section A, equation (10)), if all electrochemical potentials of a three-site chain are detuned, the zero modes should split cubically. See Extended Data Fig. 6 for theoretical simulations, Extended Data Fig. 7 for a comparison with the right two-site chain and Extended Data Fig. 8 showing the stability of three-site chain Majorana modes against the detuning of any pair of QDs. In theory, MZMs in Kitaev chains can be perfectly localized on the outermost sites, leading to no overlap and, consequently, no energy splitting. However, in practice, imperfect tuning results in finite energy splitting, which is suppressed exponentially with the number of sites in the chain (see ‘Enhanced stability’ section in the Supplementary Information). Results presented in this section are the initial steps towards achieving this exponential suppression: as the global chemical potential is detuned, the energy splitting of the MZMs decreases with increasing chain length.

**Fig. 5:**
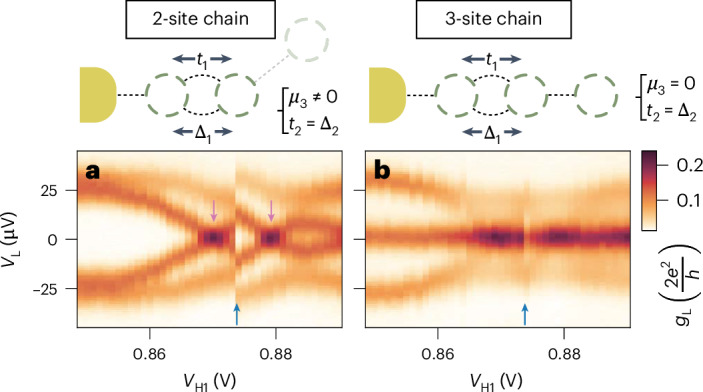
Comparison of stability against variation of *t* and Δ in a two- and three-site Kitaev chain. **a**, Conductance spectroscopy of a two-site chain as a function of *V*_H1_, which controls the magnitude of *t*_1_ and Δ_1_. QD_3_ is kept off-resonance. Pink arrows indicate the *t*_1_ = Δ_1_ sweet spot, which appears twice owing to a reproducible gate jump indicated by the blue arrow. **b**, QD_3_ is brought into resonance with the other two QDs in order to measure the spectrum of a three-site chain. Here the zero-bias conductance peak persists over the entire *V*_H1_ range >40 mV. See also Extended Data Fig. 9 for high-resolution scans around the left two-site chain sweet spot as well as the right one.

**Extended Data Fig. 9:**
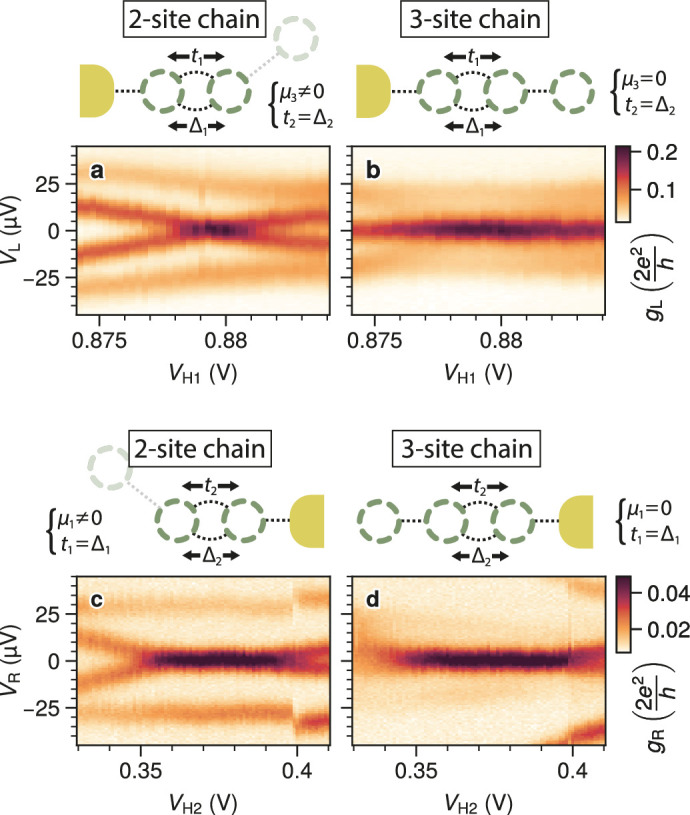
Stability against *t*_*n*_/*Δ*_*n*_ perturbations. **a, b**. Tunneling spectroscopy of a 2-site chain (panel a) and 3-site chain (panel b) as a function of *V*_H1_, measured from the left lead. This measurement is a repetition of what is presented in Fig. 5 of the main text but with higher resolution and around the *V*_H1_ ≈ 0.88 V sweet-spot. *δV*_QD3_ = − 4 mV in panel a and 0 mV in panel b. Theory simulations are reported in the Supplementary Information Fig. S4, varying only *t*_1_, only *Δ*_1_, or varying both as if a single ABS were mediating them. **c, d**. Tunneling spectroscopy of a 2-site chain (panel c) and 3-site chain (panel d) as a function of *V*_H2_. *δV*_QD1_ = − 4 mV in panel c and 0 mV in panel d. We note that the zero-bias conductance peak of panel c is more stable compared to the one of panel a, we speculate that this is due to accidental similar dispersion of *t*_2_ and *Δ*_2_ as a function of *V*_H2_ (see also Fig. S3, where higher stability regions appear for *t*_1_/*Δ*_1_ as well). Nevertheless, the 3-site zero-bias conductance peak of panel d is more persistent. We note that such peak broadens and its intensity fades at the edges of the scan, which may indicate the onset of splitting. This could be the result of imperfect centering of *V*_QD1_ at *μ*_1_ = 0.

**Extended Data Fig. 10:**
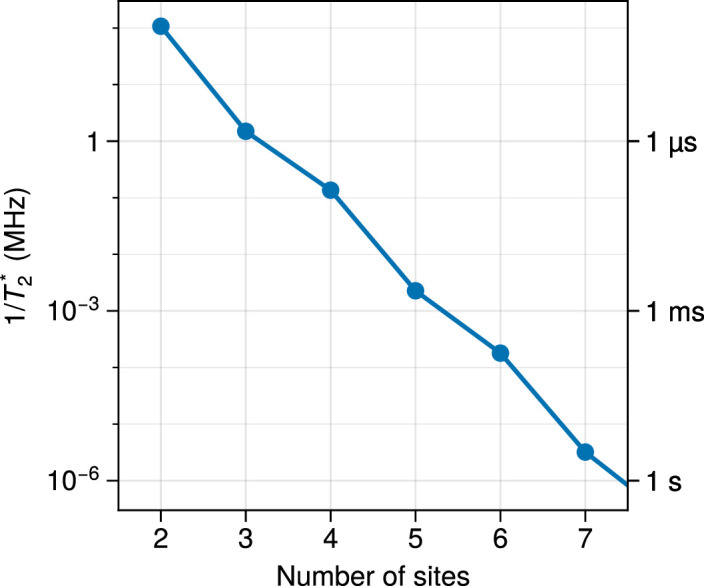
Predicted qubit coherence times as a function of the number of sites, assuming charge noise on the gates to be the only source of noise. For a fair comparison, we assume homogeneity in the Hamiltonian parameters: *t* = *Δ* = 20 *μ*eV, *α*_*D*_ = 0.04, ∂*t*/∂*V*_*H*_ = 5 × 10^−3^.

Finally, Fig. 5 compares the robustness of two- and three-site chains when leaving the pairwise sweet-spot condition of equation (2). As opposed to electrochemical potential detuning, two-site chains have no protection against tunnel coupling deviations: perturbing either *t* or Δ results in a linear splitting of the zero modes^17,19,30^. Here we reproduce such a result in panel a of Fig. 5. When QD_3_ is off-resonance, the zero bias peak of the left two-site chain is split as soon as the *V*_H1_ controlling the *t*_1_ and Δ_1_ ratio is detuned from the sweet-spot value (pink arrow). However, if we repeat the same measurement for the three-site Kitaev chain after bringing QD_3_ back on-resonance, the zero-bias conductance peak persists over the entire *V*_H1_ range (Fig. 5b), indicating tolerance to tunnel coupling deviations. We note that the *V*_H1_ range of Fig. 5 is large enough to pass through a gate jump (blue arrow), which we find reproducible across multiple scans. While gate jumps can greatly affect the spectrum of two-site chains, we find that a three-site one is robust against them. Since the zero-bias peak persists, the gate jump clearly visible in panel a becomes barely noticeable in Fig. 5b. Finally, we stress that the stability of a zero-bias peak in a two-site chain can be larger than presented in Fig. 5a. For instance, when the dispersion of *t* and Δ as a function of *V*_H_ is similar^29^, the region with *t* ≈ Δ will be extended. An example of such a scenario is shown in Extended Data Fig. 9c. The results presented in Figs. 4 and 5 demonstrate the enhanced stability of three-site chain MZMs compared with two-site chain ones. MZMs in three-site chains, while not topologically protected, are resilient against perturbations in the couplings *t*_*n*_ and Δ_*n*_ (Fig. 5), which is expected to be the main limiting factor of coherence of poor man’s Majorana-based qubits. The coherence time of a qubit made of two-site Kitaev chains was previously predicted to be ~10 ns (ref. ^30^). On the basis of the parameters extracted from the current experiment, in Methods, we estimate a qubit coherence time for a three-site Kitaev chain at *ϕ* = 0 to be around ~1 μs (we remark that the coherence time of three-site Kitaev chains without phase control is limited by the timescale of phase evolution *T*_*ϕ*_ owing to Landau–Zener^35^ transitions near gap closing). This two orders of magnitude improvement provides further motivation for developing devices with phase control. By increasing the number of sites, the protection of MZMs against perturbations of *μ*_*n*_, *t*_*n*_ and Δ_*n*_ is expected to increase further^16^. In particular, we estimate that a five-site chain would be enough for a target qubit lifetime of ~1 ms (Extended Data Fig. 10). Here we stress that the robustness of the Majorana zero energy in a three-site chain has qualitative differences from the two-site one. In the perturbative regime, that is, ∣*μ*_*n*_∣ ≪ *t*_*n*_ and ∣*t*_*n*_ − Δ_*n*_∣ ≪ *t*_*n*_, the perturbed Majorana energies *δE* are

3 $$\delta {E}_{{{2-{\rm{site}}\,{\rm{chain}}}}}=-(t-\Delta )+\frac{{\mu }_{1}{\mu }_{2}}{2t}+O({\lambda }^{3}),$$

4 $$\begin{array}{l}\delta {E}_{{{3-{\rm{site}}\,{\rm{chain}}}}}=\\-\displaystyle\frac{{\mu }_{1}({t}_{2}-{\Delta }_{2})}{2{t}_{1}}-\displaystyle\frac{{\mu }_{3}({t}_{1}-{\Delta }_{1})}{2{t}_{2}}+\displaystyle\frac{{\mu }_{1}{\mu }_{2}{\mu }_{3}}{4{t}_{1}{t}_{2}}+\displaystyle\frac{{\mu }_{1}({t}_{1}-{\Delta }_{1})({t}_{2}-{\Delta }_{2})}{4{t}_{1}^{2}}\\ +\displaystyle\frac{{\mu }_{3}({t}_{1}-{\Delta }_{1})({t}_{2}-{\Delta }_{2})}{4{t}_{2}^{2}}+O({\lambda }^{4}),\end{array}$$

where *λ* = *μ*_*n*_/*t*_*n*_ or *λ* = (*t*_*n*_ − Δ_*n*_)/*t*_*n*_ indicates the order in the perturbation expansion^36^. Note that the leading-order perturbation in a two-site chain is linear, that is, the zero energy would split linearly against deviation in coupling strength. By contrast, a three-site chain is the shortest one where no single parameter perturbation, by itself, can couple the two edge Majorana modes, split their energy and thus lead to decoherence. Here the leading-order energy splitting is quadratic owing to two different sources of perturbations. To the best of our knowledge, our work is the first to show the leading-two-order dependence of *δE*_3-site chain_ on all possible small detunings. This also explains the two-order-of-magnitude enhancement in Majorana coherence time from two- to three-site Kitaev chains (Extended Data Fig. 10). Ultimately, such robustness comes from the additional middle QD acting as the ‘bulk’ of the chain. This motivates new research directions, including longer chains, qubit experiments^37–40^ and the pursuit of new material combinations, which could provide a larger gap^41–43^.

## Conclusion

In this study, we have realized a strongly coupled three-quantum-dot chain engineered via coherent coupling of the constituent dots through crossed Andreev reflection and elastic co-tunnelling processes. Our devices have demonstrated the capability to host two adjacent two-site Kitaev chains. In addition, we illustrate that when the three dots are on-resonance, the system exhibits the spectrum expected for a three-site Kitaev chain, averaged across all possible phase differences. The set-up permits the investigation of the three-site chain MZM stability to variations in the electrochemical potential, as well as influences from crossed Andreev reflection and elastic co-tunnelling. This achievement addresses a key limitation of two-site Kitaev chains, where the finite overlap of MZM wavefunctions is considered a primary source of decoherence. In conclusion, extending Kitaev chains improves stability against *μ*_*n*_, *t*_*n*_ and Δ_*n*_, appreciated even without the phase control, the next step towards qubit experiments.

## Methods

### Nanofabrication

Our hybrid nanowire devices have been fabricated by means of the shadow-wall lithography technique thoroughly described in ref. ^44^. Specific details are described in the ‘Device structure’ paragraph of ref. ^26^ and its Supplementary Material.

### Cryogenic equipment

Transport measurements are carried in an Oxford Instruments Triton reaching a base temperature of ~20 mK. The electron temperature is estimated at ~30 mK by ref. ^45^. All electrical lines are filtered at the mixing chamber stage with a series of three RC filters, as detailed in the Supplementary Material of ref. ^26^.

### Lockin measurements

Differential conductances $({g}_{{\rm{L}}}\equiv \frac{{\mathrm{d}}{I}_{{\rm{L}}}}{{\mathrm{d}}{V}_{{\rm{L}}}},\,{g}_{{\rm{R}}}\equiv \frac{{\mathrm{d}}{I}_{{\rm{R}}}}{{\mathrm{d}}{V}_{{\rm{R}}}})$ are measured with standard lockin techniques. The raw *X* and *Y* lockin components are reported in the linked repository for all measurements. The d*V*_L_ frequency is set to 41.2999 Hz in all figures but Supplementary Fig. 2a, where it is 29 Hz, and Supplementary Figs. 6 and 7, where it is 17 Hz. The d*V*_R_ frequency is set to 31.238 Hz in all figures but Supplementary Fig. 2b, where it is 37 Hz, and Supplementary Fig. 7, where it is 21 Hz. The d*V*_L/R_ amplitude is set to 5 μV in Fig. 1 and Supplementary Figs. 2, 3a,d, 4 and 5, to 3 μV in Figs. 2, 3, 4 and 5, and Supplementary Figs. 3b,c, 10, 11, 12 and 13, and to 2 μV in Supplementary Figs. 6 and 7. This is the amplitude entering the dilution refrigerator lines; the raw locking excitation is a factor 10^4^ higher and is reduced with voltage dividers included within the room-temperature electronics^46^. The output signal is measured with ‘M1h’ current amplifiers with a 10^7^ gain^47^. There are finite background conductances *g*_L_ ≈ 0.015 2*e*^2^/*h* on the left and *g*_R_ ≈ 0.008 2*e*^2^/*h* on the right, which do not affect any of the conclusions of the paper, and remain constant. We attribute them to finite capacitive response to lockin excitations of the dilution refrigerator lines. A recent study in a similar set-up^48^, using a nominally identical refrigerator and RC filters, reported a finite background conductance of ~0.010 2*e*^2^/*h*, which is suppressed if the conductance is extracted from the numerical derivative of the measured d.c. current (see the Supporting Information of ref. ^48^). This is compatible with a finite parasitic response to the a.c. lockin excitation.

### Tuning protocol to achieve strong coupling between QDs

We report here the tuning protocol that we follow to achieve strong coupling between all QD pairs. First, we form QDs that are weakly coupled as in ref. ^26^. Weakly coupled QDs have high tunnelling barriers and sharp Coulomb diamonds, since the broadening owing to a finite lifetime is smaller than the broadening owing to temperature. Second, we start to couple the QDs more and more by progressively lowering the tunnelling barriers between them. Since, in our system, the coupling between QDs is mediated by ABSs^28,29^, to optimize the barrier height, we look at QD–ABS charge stability diagrams^30^. To optimize, for instance, the right tunnelling barrier of QD_1_, we measure the zero-bias conductance *g*_L_ as a function of *V*_QD1_ and *V*_H1_. As long as QD_1_ resonances are not affected by *V*_H1_, the tunnelling barrier is too high. So we lower the tunnelling barrier by increasing the corresponding bottom gate voltage and measure the QD_1_–ABS charge stability diagram again. When the QD resonance lines start to bend as a function of *V*_H1_, then QD_1_ and the ABS start to hybridize, indicating the onset of strong coupling. We repeat this procedure four times, once for every tunnelling barrier in between the QDs, as Extended Data Fig. 3 shows. Finally, we check that QD–QD charge stability diagrams show avoided crossings as in Fig. 1, indicating a strong coupling between each pair of QDs.

We note that our device does not have a normal-metal probe directly connected to QD_2_. Therefore, we start by tuning the middle QD, while the outer ones are not yet formed. When there is a single tunnelling barrier separating, for instance, the right hybrid and the right probe, it is possible to perform tunnelling spectroscopy of the right hybrid as Extended Data Fig. 2b shows; and it is also possible to probe QD_2_ as long as the right bias *V*_R_ is kept below the ABS energies. A possible electron transport mechanism from the right probe to QD_2_ is co-tunnelling via the ABS, or even direct tunnelling if the QD_2_ is hybridized with the ABS^49,50^. Regardless of the specific mechanism, QD_2_ can be probed from the right normal-metal lead, as panels b and c of Extended Data Fig. 3 demonstrate. After tuning the tunnelling barriers of QD_2_ with the procedure described above, we form QD_1_ and QD_3_ and tune their inner barriers in the same way, as can be seen in panels a and d of Extended Data Fig. 3. The outer tunnelling barriers, that is, the left barrier of QD_1_ and the right barrier of QD_3_, are kept high to ensure a low coupling to the normal leads.

### Poor man’s Majorana sweet spots

After achieving strong coupling between the QDs, the system needs to be tuned to the pairwise sweet-spot condition of equation (2). The procedure is similar to what is presented in ref. ^17^. The balance between crossed Andreev reflection and elastic co-tunnelling is found by looking at the direction of the avoided crossings in the QD–QD charge stability diagrams. We note that if the QDs are strongly coupled to the ABSs as in ref. ^30^, crossed Andreev reflection and elastic co-tunnelling are not well defined anymore but need to be generalized to even-like and odd-like pairings. Here we stick to the CAR/ECT nomenclature for clarity and reference further readings for the generalized concepts^30,33^. An avoided crossing along the positive diagonal indicates Δ_*n*_ dominance and an avoided crossing along the negative diagonal indicates *t*_*n*_ dominance. We select a QD_1_–QD_2_ charge degeneracy point where it is possible to range from Δ_1_ dominance to *t*_1_ dominance by varying *V*_H1_ (ref. ^29^). Similarly, we select a QD_2_–QD_3_ charge degeneracy point where it is possible to range from Δ_2_ dominance to *t*_2_ dominance by varying *V*_H2_, with the added constraint that the QD_2_ resonance must be the same for both choices. This is an important point: to be able to combine the tuning of the left and right QD pairs into a three-site chain, the gate settings of QD_2_ must be exactly the same for both pairs. To achieve this, we tune the left pair and the right pair iteratively, converging to a pairwise sweet-spot condition that shares the gate settings of QD_2_. For this reason, Figs. 2 and 3 share the same settings for all 11 bottom gates, apart, obviously, from QD_1,2,3_ depending on the panel. We note a discrepancy between the estimation of *t*_2_ = Δ_2_, which is ~40 μeV for Fig. 2 and ~60 μeV for Fig. 3. We attribute such discrepancy to a small charge jump for the right tunnelling gate of QD_2_ between the two measurements.

When crossed Andreev reflection and elastic co-tunnelling are balanced for both pairs, the charge stability diagrams show crossings instead of avoided crossings and the spectrum measured at the charge degeneracy points show zero-bias peaks (Fig. 2). Away from such sweet spots, the zero-bias peaks are split, as Fig. 5a and Extended Data Fig. 9a,c show.

### Calibration of the voltage difference between the superconducting leads

The superconducting leads of our device are separately grounded via room-temperature electronics. This facilitates the tuning and characterization of QD_2_ as shown in ref. ^26^. For a precise calibration of the voltage offset between the two superconducting leads, we tune the device to sustain a finite supercurrent (see Supplementary Fig. 2b for an example). With zero voltage drop across the device, a small voltage offset *V*_offset_ between the room-temperature grounds drops entirely through the resistances of the source and drain d.c. lines in the dilution refrigerator, ~3 kΩ each, yielding of total series resistance *R*_s_ ≈ 6 kΩ. Connecting a voltage source *V*_S1_ and a current meter *I*_S1_ to the first superconducting lead, we can calibrate the offset between the grounds using *V*_S1_ − *V*_offset_ = *R*_s_*I*_S1_. As long as there is a measurable *I*_S1_, this procedure is insensitive to the actual *R*_s_ value and is limited only by the resolution of the voltage source. Of course, even if this procedure can be very precise (see also the vertical axis of Supplementary Fig. 2b to appreciate our voltage resolution), we can expect our calibration to drift over time. This can be due, for example, to fluctuations in the room temperature and 1/*f* noise of the electronics equipment. Therefore, we measure the offset with the same precise procedure after a few days and assess how much it can drift. For the first device, such offset was always lower than 1 μV and typically closer to ~0.1 μV. For the second device, concerning only Extended Data Fig. 5 and Supplementary Fig. 2, the offset calibration was less rigorous; for Extended Data Fig. 5, we estimate an offset of ~1 μV. Lastly, we note that a finite voltage applied to the left or right normal-metal leads (*V*_L_ or *V*_R_) might lead to an effective voltage difference between the two superconducting leads owing to a voltage divider effect^51^; we calculate the impact of such effect on the voltage offset between the superconductors to be ~0.1 μV.

### Measuring the spectrum as a function of *V*_H1_

To measure the two- and three-site chain spectrum as a function of *V*_H1_ (Fig. 5), we follow the same procedure outlined for two-site chains in ref. ^30^. For every *V*_H1_ set point, we perform a sequence of three measurements:

1. We set QD_3_ off-resonance and measure the *V*_QD1_–*V*_QD2_ charge stability diagram. From the centre of the corresponding crossing (when *t*_1_ = Δ_1_) or avoided crossing (*t*_1_ ≠ Δ_1_), we extract the *δV*_QD1_ = *δV*_QD2_ = 0 charge degeneracy point.
2. We measure the two-site chain spectrum at the charge degeneracy point.
3. We set QD_3_ back on-resonance and measure the three-site chain spectrum at the charge degeneracy point.

### Theoretical model and simulation

The Hamiltonian of a three-site Kitaev chain is

5 $${H}_{K3}={\mu }_{1}{n}_{1}+{\mu }_{2}{n}_{2}+{\mu }_{3}{n}_{3}+{t}_{1}({c}_{2}^{\dagger }{c}_{1}+{c}_{1}^{\dagger }{c}_{2})+{t}_{2}({c}_{3}^{\dagger }{c}_{2}+{c}_{2}^{\dagger }{c}_{3})$$

6 $$+{\Delta }_{1}({c}_{2}^{\dagger }{c}_{1}^{\dagger }+{c}_{1}{c}_{2})+{\Delta }_{2}({e}^{i\phi }{c}_{3}^{\dagger }{c}_{2}^{\dagger }+{e}^{-i\phi }{c}_{2}{c}_{3}).$$

Here *c*_*i*_ is the annihilation operator of the orbital in dot *i*, ${n}_{i}={c}_{i}^{\dagger }{c}_{i}$ is the occupancy, *μ*_*i*_ is the orbital energy relative to the superconductor Fermi energy, *t*_*i*_ and Δ_*i*_ are the normal and superconducting tunnellings between dots *i* and *i* + 1, and *ϕ* is the phase difference between the two superconducting leads. Physically, *t*’s and Δ’s are the elastic co-tunnelling and crossed Andreev reflection amplitudes mediated by the subgap ABSs in the hybrid segments. In the Nambu basis, the above Hamiltonian can be written as

7 $$\begin{array}{rcl}&&H=\frac{1}{2}{\Psi }^{\dagger } {h}_{BdG} \Psi ,\\ &&\Psi ={\left({c}_{1},{c}_{2},{c}_{3},{c}_{1}^{\dagger },{c}_{2}^{\dagger },{c}_{3}^{\dagger }\right)}^{T},\\ &&{h}_{BdG}=\left(\begin{array}{cccccc}{\mu }_{1}&{t}_{1}&0&0&-{\Delta }_{1}&0\\ {t}_{1}&{\mu }_{2}&{t}_{2}&{\Delta }_{1}&0&-{\Delta }_{2}{e}^{i\phi }\\ 0&{t}_{2}&{\mu }_{3}&0&{\Delta }_{2}{e}^{i\phi }&0\\ 0&{\Delta }_{1}&0&-{\mu }_{1}&-{t}_{1}&0\\ -{\Delta }_{1}&0&{\Delta }_{2}{e}^{-i\phi }&-{t}_{1}&-{\mu }_{2}&-{t}_{2}\\ 0&-{\Delta }_{2}{e}^{-i\phi }&0&0&-{t}_{2}&-{\mu }_{3}\end{array}\right).\end{array}$$

When the system is coupled to normal leads, the scattering matrix describing the transmission and reflection amplitudes between modes in the leads can be expressed by the Weidenmuller formula

8 $$S(\omega )=1-i{W}^{\;\dagger }{\left(\omega -{h}_{BdG}+\frac{i}{2}W{W}^{\;\dagger }\right)}^{-1}W,$$

where the tunnel matrix *W* is defined as

9 $$W=\,\text{diag}\,\left(\sqrt{{\Gamma }_{\mathrm{L}}},0,\sqrt{{\Gamma }_{\mathrm{R}}},-\sqrt{{\Gamma }_{\mathrm{L}}},0,-\sqrt{{\Gamma }_{\mathrm{R}}}\right),$$

with *Γ*_L/R_ being the dot–lead coupling strength on the left and right ends, respectively. At zero temperature, the differential conductance is expressed as

10 $${G}_{ij}^{(0)}(\omega )\equiv \mathrm{d}{I}_{i}/\mathrm{d}{V}_{j}={\delta }_{ij}-| {S}_{ij}^{ee}(\omega ){| }^{2}+| {S}_{ij}^{he}(\omega ){| }^{2}$$

in unit of *e*^2^/*h*. Here *i*, *j* = 1, 2, 3, and *ω* denotes the bias energy in the leads. The finite-temperature conductance is obtained by a convolution between the zero-temperature one and the derivative of the Fermi distribution

11 $${G}_{ij}^{T}(\omega )=\mathop{\int}\nolimits_{\!\!-\infty }^{+\infty }dE\frac{{G}_{ij}^{(0)}(E\;)}{4{k}_{\mathrm{B}}T{\cosh }^{2}[(E-\omega )/2{k}_{\mathrm{B}}T\;]}.$$

In performing the numerical simulations, we choose the coupling strengths to be *t*_1_ = Δ_1_ = 10 μeV, *t*_2_ = Δ_2_ = 30 μeV based on the positions of the excited states shown in Fig. 3. The electron temperature in the normal leads, *T* ≈ 35 mK, corresponds to a broadening *k*_B_*T* ≈ 3 μeV. The strengths of the lead–dot couplings are chosen to be *Γ*_L_ = 1.5 μeV and *Γ*_R_ = 0.3 μeV, such that the conductance values obtained in the numerical simulations are close to those in the experimental measurements. Moreover, to capture the effects of lever arms strength differences in the three dots, we choose *δμ*_1_ = *δμ*, *δμ*_2_ = *δμ*, *δμ*_3_ = 0.3*δμ*. Crucially, we notice that in the particular experimental devices studied in this work, since the voltage bias between the two superconducting leads cannot be set to zero precisely, 0.1 μV ≲ *δV* ≲ 1 μV, the phase difference precesses with periods of $2\,{\rm{ns}}\lesssim {T}_{\phi } \approx \frac{h}{2e\delta V}\lesssim 20\,{\rm{ns}}$. However, the lifetime of an electron spent in a QD is at the order of *τ*_*e*_ ≈ *ℏ*/*Γ* ≈ 1 ns. This is the timescale of a single event of electron tunnelling giving electric current, which would take a random value of phase difference *ϕ* since *τ*_*e*_ is smaller than or of similar order as the period of the phase winding *T*_*ϕ*_. However, both *τ*_*e*_ and *T*_*ϕ*_ are a very small timescale relative to the d.c. current measurement time (~1 s). Therefore, any particular data point collected in the conductance measurement is an average over ~10^9^ tunnelling events with different possible phases. Theoretically, we capture this effect by performing a phase average on the differential conductance as follows:

12 $${\langle {G}_{ij}^{T}(\omega )\rangle }_{\phi }\equiv \mathop{\int}\nolimits_{\!\!0}^{2\pi }\frac{d\phi }{2\pi }{G}_{ij}^{T}(\omega ,\phi ).$$

The numerically calculated conductances shown in the main text are obtained by averaging over 50 values of phases evenly distributed between 0 and 2*π*.

### Estimation of dephasing rate for the Kitaev chain qubit

In this subsection, we perform a numerical estimation of the dephasing time of different types of Kitaev chain qubit, similar to ref. ^30^ in spirit. In particular, we consider three different types of Kitaev chain qubit: two-site Kitaev chain with weak and strong dot–hybrid coupling, and three-site Kitaev chain with a fixed phase difference *ϕ* = 0. A qubit consists of two copies of Kitaev chains, ${H}_{K}^{A}$ and ${H}_{K}^{B}$, respectively. Without loss of generality, we focus on the subspace of total parity even, and therefore the two qubit states are defined as $| 0\left.\right\rangle =| {e}_{A},{e}_{B}\left.\right\rangle$ and $| 1\left.\right\rangle =| {o}_{A},{o}_{B}\left.\right\rangle$, where $| o\left.\right\rangle$ and $| e\left.\right\rangle$ denote the odd- and even-parity ground states in each chain and $| {e}_{A},{e}_{B}\left.\right\rangle \equiv {| e\left.\right\rangle }_{A}\otimes {| e\left.\right\rangle }_{B}$ is the tensor state. Note that here we do not consider inter-chain coupling, which depends on the device details that have not been implemented so far, thus going beyond the scope of this work. Therefore, our estimation only provides an upper limit of the dephasing time in a Majorana qubit. Furthermore, we assume that charge noise within a Kitaev chain is the main source of decoherence in the device that we consider here. As such, the energy difference between the two qubit states would fluctuate, giving rise to a dephasing rate $1/{T}_{2}^{\;* } \approx \delta E/\hslash$, where *δE* is the characteristic energy splitting of *E*_*oo*_ − *E*_*ee*_. Generally, charge noise is dominated by fluctuations of charge impurities in the environment. However, as shown in ref. ^52^, the charge impurity fluctuations can be equivalently described by fluctuations in the gate voltages. Theoretically, the voltage fluctuations enter the Kitaev chain Hamiltonian as follows:

13 $$\begin{array}{rcl}\delta {\mu }_{i}&=&{\alpha }_{i} \delta {V}_{i},\\ \delta {t}_{j}&=&\frac{\partial {t}_{j}}{\partial {V}_{{H}_{j}}} \delta {V}_{j},\end{array}$$

with *α*_*i*_ being the lever arm of the *i*th QD. In the second formula, the derivative is extracted from a single pair of poor man’s Majoranas (Fig. 5a). We emphasize that the fluctuations of *t*_*j*_ and Δ_*j*_ are correlated because both of them are induced by the ABS in the hybrid, which is controlled by a single electrostatic gate.

Here, as a first-order approximation, we assume that the fluctuations are on *t*_*j*_ while Δ_*j*_ remains constant. Charge noise is also known as slow-varying in time and thus can be well described with a quasi-static disorder approximation (see Ref. ^53^). We generate 5,000 different disorder realizations of the set of gate voltages. Moreover, we assume that two chains in a qubit are subject to independent sources of charge noises and thus we can calculate their energy splitting individually and the energy splitting of the qubit states is just the sum as ${E}_{oo}-{E}_{ee}=({E}_{o}^{A}-{E}_{e}^{A})+({E}_{o}^{B}-{E}_{e}^{B})$. Finally, we take the standard deviation of ${\langle {E}_{oo}-{E}_{ee}\rangle }_{\mathrm{std}}$, which eventually gives the dephasing rate.

The voltage fluctuations obey Gaussian distribution with mean zero and standard deviation *δV* ≈ 10 μeV, as discussed in a similar experimental device^30^. In our models of Kitaev chains, we consider independent fluctuation sources in dots and in the hybrid segment. Our analysis considers three distinct scenarios: dephasing owing to dot energies only, hybrid coupling only and both of them. The device parameters used in our numerical simulations and the results of the estimations are summarized in Table 1. In Extended Data Fig. 10, we show how the estimated dephasing time ${T}_{2}^{* }$ scales with the number of chain sites. For a fair comparison, we now choose the model parameters (for example, *t*_*i*_ = Δ_*i*_ = 20 μeV and lever arms *α*_*D*_ = 0.04*e*) to be identical for all *N*.

**Table 1:** Estimation of dephasing rate for different types of Kitaev chain qubit, assuming charge noise to be the only source of noises

Device parameters	QD-PMM^17^	YSR-PMM^30^	Kitaev-3 (*ϕ* = 0)
*α*_QD_ [*e*]	0.3	0.04	0.04
∂*t*/∂*V*_H_ [*e*]	5 × 10^−3^	5 × 10^−3^	5 × 10^−3^
*t*, Δ [μeV]	10	40	10 (left), 30 (right)
$1/{T}_{2}^{* }\,[{\rm{MHz}}]$ (*μ* noises)	~900	~4	~0.1
$1/{T}_{2}^{* }\,[{\rm{MHz}}]$ (*t* noises)	~100	~100	0
$1/{T}_{2}^{* }\,[{\rm{MHz}}]$ (all noises)	~900	~100	~2

YSR abbreviates 'Yu-Shiba-Rusinov' states^30^.

## Online content

Any methods, additional references, Nature Portfolio reporting summaries, source data, extended data, supplementary information, acknowledgements, peer review information; details of author contributions and competing interests; and statements of data and code availability are available at 10.1038/s41565-025-01894-4.

## Supplementary information

Supplementary Information Supplementary Figs. 1–4, 4 sections on the details of theoretical models supported by the discussion in the main text.

## Data Availability

All raw data in the publication, the code used to generate the figures, and the code used for the theory calculations are available at 10.5281/zenodo.13891286 (ref. ^54^).
