# Supplementary material for: Enhanced Majorana stability in a three-site Kitaev chain
Source: Nat Nanotechnol. 2025 Mar 31;20(6):726–31. doi: 10.1038/s41565-025-01894-4 (PMC12181078; doi:10.1038/s41565-025-01894-4)
Supplement: Supplementary file 1 — Supplementary Figs. 1–4, 4 sections on the details of theoretical models supported by the discussion in the main text. [file 41565_2025_1894_MOESM1_ESM.pdf]

nature nanotechnology

Supplementary information https://doi.org/10.1038/s41565-025-01894-4

Enhanced Majorana stability in a three-site Kitaev chain

In the format provided by the authors and unedited

CONTENTS

A. Enhanced Stability 2

B. Derivation of the effective three-site Kitaev chain model 4

C. Energies and wavefunctions of Majorana modes at phase ϕ 6

D. Comparison with recent theoretical findings 9

Supplementary Figures 10

S1 10

S2 11

S3 12

S4 13

References 14

A. Enhanced Stability

For Majorana zero modes at the sweet spot of an N-site Kitaev chain ($t_i = \Delta_i$, $\mu_i = 0$), the energy deviation due to onsite chemical potential fluctuations can be expressed as:

$$\delta E_{K_N} \equiv E_{\text{odd,gs}} - E_{\text{even,gs}} = \mu_N \prod_{i=1}^{N-1} \frac{\mu_i}{2t_i}, \tag{1}$$

where $t_i = \Delta_i$ are the strengths of the normal and superconducting couplings between sites i and $i+1$.For a two-site Kitaev chain, this reduces to:

$$\delta E_{K_2} = \frac{\mu_1 \mu_2}{2t} = C_{K_2} \cdot \tilde{\mu}^2, \tag{2}$$

where $\mu_i = \mu$ and $\tilde{\mu} = \mu/(1\,\mu\text{eV})$ is dimensionless. The quadratic dependence on μ indicates quadratic protection against chemical potential fluctuations in a two-site chain. Here, C_{K_2} has units of μeV and represents the energy deviation when both dot orbitals are detuned from the Fermi energy by 1 μeV. Using experimentally measured values of $t_1 = 10\,\mu\text{eV}$ and $t_2 = 30\,\mu\text{eV}$, we estimate $C_2 = 5 \times 10^{-2}\,\mu\text{eV}$ and $1.66 \times 10^{-2}\,\mu\text{eV}$ for the left and right pairs of Poor Man's Majorana modes, respectively. To further analyze the energy spectrum, we consider the effective Hamiltonian of a 2-site Kitaev chain:

$$H_{\text{even}} = \begin{pmatrix} 0 & \Delta \\ \Delta & \varepsilon_L + \varepsilon_R \end{pmatrix}, \quad H_{\text{odd}} = \begin{pmatrix} \varepsilon_L & t \\ t & \varepsilon_R \end{pmatrix} \tag{3}$$

in the basis of $|00\rangle, |11\rangle$ and $|10\rangle, |01\rangle$ for even- and odd-parity subspaces, respectively. The eigenenergies are:

$$E_{e,\pm} = \frac{\varepsilon_L + \varepsilon_R}{2} \pm \sqrt{\Delta^2 + \left(\frac{\varepsilon_L + \varepsilon_R}{2}\right)^2}, \tag{4}$$

$$E_{o,\pm} = \frac{\varepsilon_L + \varepsilon_R}{2} \pm \sqrt{t^2 + \left(\frac{\varepsilon_L - \varepsilon_R}{2}\right)^2}. \tag{5}$$

At the sweet spot where $t = \Delta$ and $\varepsilon_L = \varepsilon_R = \delta\varepsilon$, the excitation energies are:

$$E_{1,2} = E_{o,\pm} - E_{e,-} = \pm t + \sqrt{t^2 + (\delta\varepsilon)^2} \tag{6}$$

We can analyze this in different regimes:

1. For small detuning ($\delta\varepsilon \ll t$), the first excited energy is approximately:

$$E_1 \approx \frac{(\delta\varepsilon)^2}{2t} \tag{7}$$

showing a quadratic dispersion as a function of $\delta\varepsilon$.

2. For large detuning ($\delta\varepsilon \gg t$), we have:

$$E_1 \approx \delta\varepsilon - t \tag{8}$$

which is a linear dispersion in $\delta\varepsilon$.

3. For all values of $\delta\varepsilon$, the energy gap between the first and second excited states remains fixed at:

$$E_2 - E_1 = 2t. \tag{9}$$

These results demonstrate how the energy spectrum of a 2-site Kitaev chain responds to chemical potential fluctuations in different regimes, highlighting the quadratic protection for small detunings and the transition to linear behavior for large detunings.

For an extended three-site Kitaev chain, the energy deviation becomes

$$\delta E_{K_3} = \frac{\mu_1\mu_2\mu_3}{4t_1t_2} = C_{K_3} \cdot \tilde{\mu}^3, \tag{10}$$

which is a cubic protection. Now $C_{K_3} = 8.33 \times 10^{-4}\ \mu eV$, which is smaller than C_{K_2} by two orders of magnitudes, indicating a significantly enhanced degree of stability in the three-site Kitaev chain than its two-site version. Moreover, we illustrate the physical meaning of "exponential protection" in scaling up a Kitaev chain. Without loss of generality, assuming homogeneity of the model parameters ($t_i = \Delta_i = t \equiv E_g/2$ with E_g being the energy gap), we have

$$\delta E_{K_N} = \mu_N \prod_{i=1}^{N-1} \frac{\mu_i}{2t_i} = \frac{\mu^N}{E_g^{N-1}} = E_g \left(\frac{\mu}{E_g}\right)^N = E_g \exp\{-N \log(E_g/\mu)\}. \tag{11}$$

Physically, it means that when the onsite energies of all the N dots are detuned from zero by the same amount $\mu \ll E_g$, the energy splitting of the Majorana zero modes will decrease exponentially fast with the increasing number of sites at a rate of $\log(E_g/\mu)$.

B. Derivation of the effective three-site Kitaev chain model

In this section, we derive the effective Hamiltonian of a three-site Kitaev chain from a more microscopic level. For the three quantum dots, in the presence of large Zeeman spin splitting and Coulomb interaction, we can approximate it to a single spin-polarized orbital as below:

$$H_{Di} = \mu_i(n_{i\uparrow} + n_{i\downarrow}) + 2E_Z n_{i\uparrow} + U_D n_{i\uparrow} n_{i\downarrow} \approx \mu_i n_{i\downarrow}, \tag{12}$$

for $i = 1, 2, 3$ and $\mu_i \approx 0$. Here in the derivation, we focus on the spin-down orbitals in all dots, but the analysis holds for other spin polarizations as well. On the other hand, the hybrid segment hosts subgap Andreev Bound States, of which the Hamiltonian is

$$H_{Aj} = \mu_j(n_{j\uparrow} + n_{j\downarrow}) + |\Delta_j^{\text{ind}}|(e^{i\phi_j} c_{j\uparrow}^\dagger c_{j\downarrow}^\dagger + e^{-i\phi_j} c_{j\downarrow} c_{j\uparrow}) \tag{13}$$

where $j = L, R$, and $|\Delta_j^{\text{ind}}|$ is the magnitude of the induced gap, and ϕ_j is the superconducting phase. The coupling between the dot and the hybrid is described by the following tunnel Hamiltonian

$$H_{tunn} = \sum_{i=1,2} \sum_{\sigma=\uparrow,\downarrow} \left(w c_{i\sigma}^\dagger d_{i\sigma} + \sigma w_{so} c_{i\bar{\sigma}}^\dagger d_{i\sigma} + w d_{i+1\sigma}^\dagger c_{i\sigma} + \sigma w_{so} d_{i+1\bar{\sigma}}^\dagger c_{i\sigma} \right) + h.c., \tag{14}$$

where w and w_{so} are the tunneling amplitudes for spin-conserving and spin-flipping processes. As shown in Ref. [1], in the tunneling regime, i.e., $w, w_{so} \ll |\Delta^{\text{ind}}|$, the Andreev bound states in the hybrid will mediate normal and superconducting couplings of quantum dots via elastic cotunneling and crossed Andreev reflection processes, giving

$$\begin{aligned} t_1 &= (w^2 - w_{so}^2)\frac{u_L^2 - v_L^2}{E_{AL}}, \\ \Delta_1 &= w w_{so} \frac{2u_L v_L}{E_{AL}} e^{i\phi_L}, \\ t_2 &= (w^2 - w_{so}^2)\frac{u_R^2 - v_R^2}{E_{AR}}, \\ \Delta_2 &= w w_{so} \frac{2u_R v_R}{E_{AR}} e^{i\phi_R}, \end{aligned} \tag{15}$$

where u_j, v_j are the BCS coherence factors of the ABS, and $E_{Aj} = \sqrt{\mu_j^2 + (\Delta_j^{\text{ind}})^2}$ is the excitation energy. Thereby, by varying the chemical potential of the ABS, we can obtain the sweet spot by balancing the normal and superconducting coupling strengths ($|t_i| = |\Delta_i|$).

Furthermore, by performing a gauge transformation on the dot orbitals, we can remove the possible phases in three couplings in Eq. (15), and obtain

$$\begin{aligned}
&t_1 \to |t_1|, \quad \Delta_1 \to |\Delta_1|, \\
&t_2 \to |t_2|, \quad \Delta_2 \to |\Delta_2| e^{i\phi}, \\
&\phi = \phi_R - \phi_L + \arg(t_1) + \arg(t_2).
\end{aligned} \tag{16}$$

That is, the effect of the phase difference between the two superconducting leads is now completely absorbed in a single parameter ϕ. We therefore justify the use of the effective Hamiltonian in Eq. (17) as the low-energy description of the dot-hybrid array.

$$\begin{aligned}
H_{K3} = \mu_1 n_1 + \mu_2 n_2 + \mu_3 n_3 + t_1(c_2^\dagger c_1 + c_1^\dagger c_2) + t_2(c_3^\dagger c_2 + c_2^\dagger c_3) \\
+ \Delta_1(c_2^\dagger c_1^\dagger + c_1 c_2) + \Delta_2(e^{i\phi} c_3^\dagger c_2^\dagger + e^{-i\phi} c_2 c_3).
\end{aligned} \tag{17}$$

We emphasize that in performing numerical simulations of dot energy detuning, as shown in Figs. 3 and 4, the couplings between dots are just denoted by t_j, Δ_j, while when considering the effect of voltage change in the hybrid segment for Fig. S4e-f, we reintroduce the effect of ABS using Eq. (15) for the couplings in order to capture the μ_A dependence features.

C. Energies and wavefunctions of Majorana modes at phase ϕ

In this subsection, we calculate the wavefunctions of Majorana zero modes in a three-site Kitaev chain with an arbitrary phase. The Bogoliubov-de-Gennes Hamiltonian is the following:

$$
\begin{aligned}
&H = \frac{1}{2}\Psi^\dagger \cdot h_{BdG} \cdot \Psi, \\
&\Psi = (c_1, c_2, c_3, c_1^\dagger, c_2^\dagger, c_3^\dagger)^T, \\
&h_{BdG} = \begin{pmatrix} \mu_1 & t_1 & 0 & 0 & -\Delta_1 & 0 \\ t_1 & \mu_2 & t_2 & \Delta_1 & 0 & -\Delta_2 e^{i\phi} \\ 0 & t_2 & \mu_3 & 0 & \Delta_2 e^{i\phi} & 0 \\ 0 & \Delta_1 & 0 & -\mu_1 & -t_1 & 0 \\ -\Delta_1 & 0 & \Delta_2 e^{-i\phi} & -t_1 & -\mu_2 & -t_2 \\ 0 & -\Delta_2 e^{-i\phi} & 0 & 0 & -t_2 & -\mu_3 \end{pmatrix}.
\end{aligned}
\tag{18}
$$

The goal is to find the Majorana wavefunctions at the sweet spot $\mu_n = 0, t_n = \Delta_n$, but with an arbitrary value of ϕ. Since Majoranas are defined as self-adjoint zero-energy quasiparticles, they obey

$$\gamma = \sum_{n=1}^{3} (\xi_n c_n + \xi_n^* c_n^\dagger), \tag{19}$$

where ξ_n is the Majorana wavefunction on site-n and correspondingly, $\rho_n = |\xi_n|^2$ is the wavefunction density. By solving

$$H_{BdG}\psi = 0, \tag{20}$$

where $\psi = (\xi_1, \xi_2, \xi_3, \xi_1^*, \xi_2^*, \xi_3^*)^T$, it is straightforward to show that the wavefunctions of two Majorana zero modes are:

$$
\begin{aligned}
&\psi_1 = (i, 0, 0, -i, 0, 0)^T/\sqrt{2}, \\
&\psi_2 = (0, 0, e^{i\phi/2}, 0, 0, e^{-i\phi/2})^T/\sqrt{2},
\end{aligned}
\tag{21}
$$

for an arbitrary phase ϕ. Here a different value of ϕ only changes ψ_2 via a gauge-dependent phase. By contrast, the wavefunction densities of both Majorana modes are phase independent. For example, the density distribution of ψ_1 is $\rho_1 = \frac{1}{2}, \rho_2 = 0, \rho_3 = 0$, i.e., the

Majorana is completely localized at QD_1. For ψ_2 it is $\rho_1 = 0, \rho_2 = 0, \rho_3 = \frac{1}{2}$, which is completely localized in QD_3. Therefore the density distributions of the two Majorana zero modes are phase-independent. However, at a special phase of $\phi = \pi$, there exist two additional zero-energy solutions as below

$$\psi_3 = (0,1,0,0,1,0)^T/\sqrt{2},$$
$$\psi_4 = (t_2, 0, -t_1, t_2, 0, -t_1)^T/\sqrt{2(t_1^2+t_2^2)}. \tag{22}$$

Here the density distribution of ψ_3 is $\rho_1 = 0, \rho_2 = \frac{1}{2}, \rho_3 = 0$, which is completely localized in QD_2. By contrast ψ_4 is a delocalized zero-energy state which has wavefunction densities on both QD_1 and QD_3, i.e., $\rho_1 = \frac{t_2^2}{2(t_1^2+t_2^2)}, \rho_2 = 0, \rho_3 = \frac{t_1^2}{2(t_1^2+t_2^2)}$. We thus conclude that the zero-bias conductance peak at the sweet spot ($\mu_n = 0, t_n = \Delta_n$) is induced by an isolated Majorana zero modes regardless of the uncertainty in phase ϕ. The only exception is $\phi = \pi$, where an additional pair of zero modes appear, making the three-site chain gapless.

Now we consider parameter regions away from the sweet spot, i.e., one of the three quantum dot is detuned, in order to understand the conductance spectroscopies shown in Fig. 3. We first consider detuning QD_1. In the vicinity of 0-phase, i.e., $\phi \sim 0$, the MZM on QD_3 γ_2 is unaffected, while the wavefunction of γ_1 becomes

$$\psi_1 = \left(\frac{i}{\sqrt{1+v^2}}, \frac{-vi}{\sqrt{1+v^2}}, 0, \frac{-i}{\sqrt{1+v^2}}, \frac{vi}{\sqrt{1+v^2}}, 0\right)^T/\sqrt{2}, \tag{23}$$

where $v = \mu_1/(2t_1\cos(\phi/2))$ for $\phi \sim 0$. Thus the Majorana wavefunction densities of ψ_1 are $\rho_1 = \frac{1}{2(1+v^2)}, \rho_2 = \frac{v^2}{2(1+v^2)}, \rho_3 = 0$, i.e., part of the wavefunction of ψ now leaks into QD_2. On the other hand, in the vicinity of π-phase, i.e., $\phi \sim \pi$, detuning μ_1 would gap out ψ_1 and ψ_3 because both zero modes have a finite wavefunction on QD_1. The energy splitting is $E = \frac{t_2}{\sqrt{t_1^2+t_2^2}} \times \mu_1$, as shown in Ref. [2]. The wavefunction calculations thus provides an intuitive understanding of the measured conductance spectroscopy shown in Fig. 3. We find:

1. Since detuning QD_1 does not affect the wavefunction of ψ_2 which is completely localized at QD_3, the ZBCP measured from the right lead therefore remains stable as a function of V_{QD1}, as shown in Fig. 3d.

2. In the vicinity of 0-phase, detuning QD_1 decreases the wavefunction density of ψ_1 on QD_1. Therefore the ZBCP measured from the left lead, although remain at zero bias, is suppressed as a function of V_{QD1}, as shown in Fig. 3a.

3. In the vicinity of π-phase, detuning QD_1 gaps out ψ_1 and ψ_4 on QD_1. Therefore the ZBCP measured from the left lead splits as a function of V_{QD1}, as shown in Fig. 3a.

We emphasize that here we simultaneously observed features 2 and 3 in Fig. 3a because the measured conductance is averaged over all phase differences. In addition, the underlying physics of detuning QD_3 is identical to that of detuning QD_1, except that now the roles of (G_L, G_R), (μ_1, μ_2), and (t_1, t_2) in the analysis should be interchanged.

Next, we consider detuning of QD_2. In the vicinity of $\phi \sim 0$, the wavefunctions of ψ_1 and ψ_2 both remain unaffected by such a detuning, because they are completely localized on outer dots. Around $\phi \sim \pi$, ZBCP also remain the same, because no energy splitting happens as for μ_1 detuning, since all the four zero modes $\psi_{1,2,3,4}$ have no wavefunction overlap on QD_2. We thus see a robust ZBCP for small detuning of QD_2 from both left and right conductance. However, when the middle dot is detuned faraway (for whatever value of ϕ), the middle dot becomes a vacuum, and the three-site chain asymptotically behaves like two disconnected normal dots on outer sites. This explains why a conductance peak from the excited states goes down to zero bias as V_{QD2} becomes large, merging with the MZM-induced zero-bias peak. Thus the observed ZBCP is simply a conductance for a normal dot orbital on resonance.

D. Comparison with recent theoretical findings

Recent theoretical works noticed that scaling up to longer chains may not always lead to increased Majorana protection in certain ranges of parameters [3–5]. In our work, we always observe increased zero-bias peak stability in three-site chain configuration. We attribute this to the large InSb g-factor, which ensures the chain is close to the Kitaev limit: $E_z \gg k_B T, t_n, \Delta_n$. Future works may investigate a lower E_z regime and assess whether this ideal behavior breaks down.

For instance, in ref. [4] as well as in ref. [6], it is found that next-nearest-neighbor tunnelings (t_{nn}) can emerge in a three-site Kitaev chain due to higher-order electron tunneling, which is detrimental to the Majorana protection at the finely tuned sweet spot. The ratio of the next-nearest-neighbor tunnelings to the nearest-neighbor ones ($t_i = \Delta_i$) is mainly determined by $t_{nn}/t_i \sim O(t_i/E_Z)$, where E_Z is the Zeeman spin splitting in normal quantum dots. By contrast, such a correction term does not exist in a minimal two-site version. Therefore, when t_{nn}/t_i becomes evident, the three-site chain may behave worse than a two-site system, as claimed in ref. [4].

In the device studied in the current work, since the effective couplings $t \approx (t_1 + t_2)/2 \approx 20\mu\text{eV}$ are much weaker than the Zeeman energy $E_Z \approx 500\mu\text{eV}$, next-nearest neighbor tunnelings would be as small as ~ 0.8 μeV, which is much smaller than the excitation gap as well as the conductance peak broadening. We thus conclude that our dot-hybrid chain device is in the strong Zeeman spin splitting regime and a spinless Kitaev chain is a good description of the low-energy physics.

SUPPLEMENTARY FIGURES

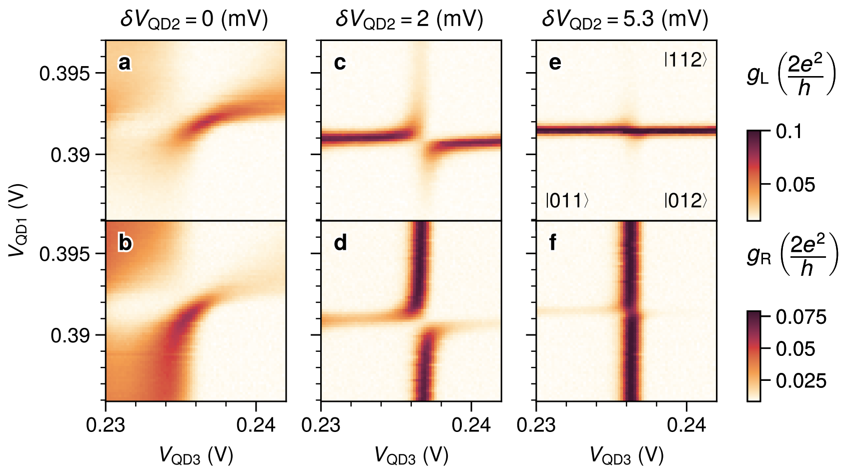

Fig. S1. **Impact of** QD_2 **on the coupling between the outer dots. a-b.** QD_1–QD_3 charge stability diagrams measured from the left probe (panel a) and the right one (panel b). At the center of the avoided crossing, all QDs are on resonance. **c-d.** Same measurements of panels a and b, but with QD_2 2 mV off-resonance. **e-f.** Here QD_2 is 5.3 mV off-resonance. The more QD_2 is detuned, the smaller the QD_1–QD_3 avoided crossings are. In panels e and f, the avoided crossings are barely noticeable, indicating suppression of the coupling between the outer dots. Finally, we note that not only the size of the avoided crossings but also the amount of conductance indicates suppression of the QD_1–QD_3 coupling: in panel e, the vertical conductance line representing the QD_3 charge transition is barely visible; while in panel f it is the horizontal line corresponding to the QD_1 transition to be suppressed.

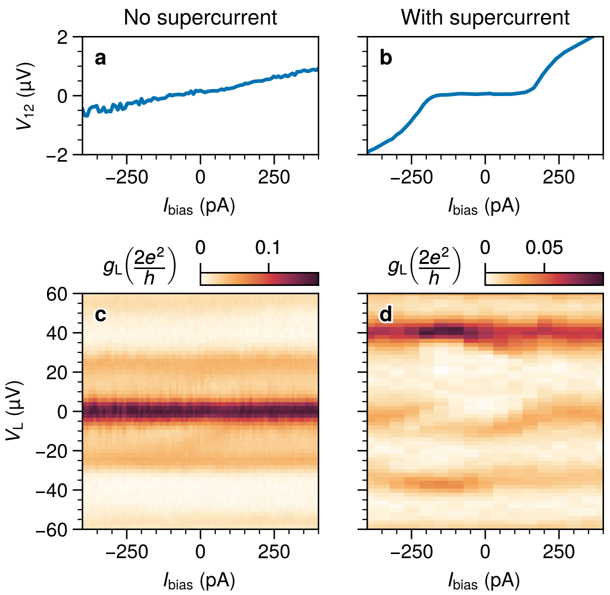

Fig. S2. **Impact of a finite supercurrent between the two superconducting leads. a, b.** I-V curves without and with a measurable supercurrent. The voltage V_{12} between the two superconductors is measured as a function of the current bias I_{bias} between them. See the code in the shared repository for measurement details. **c, d.** Left spectroscopy of a three-site chain as a function of I_{bias}, without and with a measurable supercurrent. To avoid complications due to supercurrent, in all the measurements reported in this manuscript (apart from Fig. S2b,d) the tunneling barriers forming QD_2 are kept high enough to suppress the supercurrent. In the left column of this figure, we check that such settings show a linear I-V curve and that the I_{bias} doesn't affect the three-site chain spectrum. To prove that our device can carry supercurrent and this might affect the spectrum of a three-site chain, we lower the QD_2 tunneling barriers and measure what is presented in panels b and d. A detailed investigation of the effects of supercurrent is beyond the scope of this manuscript and is left for follow-up works.

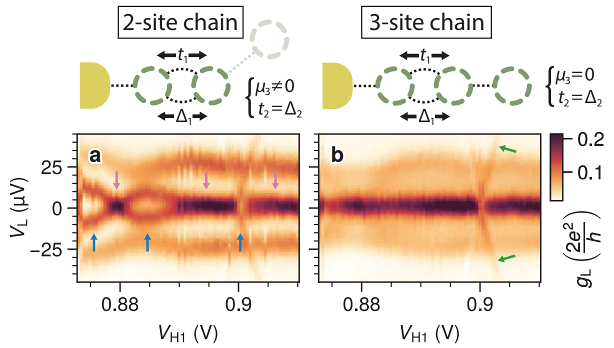

Fig. S3. **Stability against t_1 and Δ_1 variations in a large range. a, b.** Left tunneling spectroscopy of a 2-site chain (panel a) and 3-site chain (panel b) as in Fig. 5 and ED9 but for a different V_{H1} range. Here we can appreciate that even two-site chains can have improved the zero-bias conductance peak stability: the pink arrows highlight three sweet-spot regions, two of which have a more stable zero-bias peak. This can be due for instance to accidentally similar dispersion of t_1 and Δ_1 as a function of V_{H1}, which can in principle be optimized using the external magnetic field direction [7]. However, this requires careful tuning or searching to avoid non-sweet-spot regions, highlighted here with blue arrows. The 3-site chain has instead a stable zero-bias peak over the full range (panel b). We also note, for both panels, the presence of a spurious resonance (green arrows) likely due to an accidental dot near the left probe.

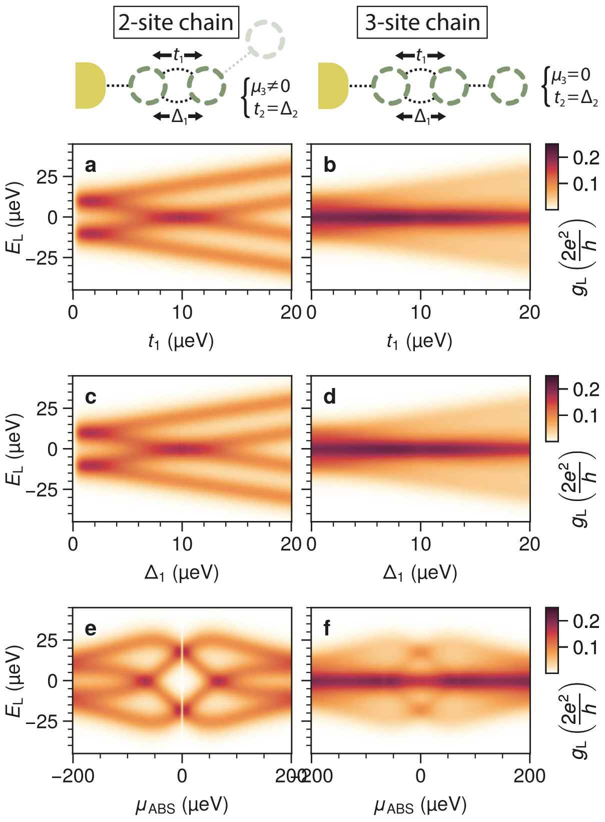

Fig. S4. **Simulation of the stability against t_1 and Δ_1 variations. a, b.**. Conductance simulation with t_1 varied from 0 to 20 µeV for a 2-site chain (panel a) and a 3-site chain (panel b) exactly as Fig. 5c,d. $\Delta_1 = 10$ µeV. **c, d.**. Conductance simulations as a function of Δ_1. $t_1 = 10$ µeV. The result is identical to the panels above where t_1 was varied instead. **e, f.**. Conductance simulations in a more realistic scenario, where t_1 and Δ_1 are varied simultaneously as if there were a single ABS mediating them [1, 7]. In all scenarios, the left column – corresponding to 2-site chains – exhibits zero energy crossings, while the right column – corresponding to 3-site chains – shows persistent zero-bias peaks over the full range. For 3-site chain simulations, the conductance is averaged over 50 phase values of Δ_2, uniformly distributed from 0 to 2π.

[1] C.-X. Liu, G. Wang, T. Dvir, and M. Wimmer, Tunable superconducting coupling of quantum dots via andreev bound states in semiconductor-superconductor nanowires, Physical Review Letters **129**, 10.1103/physrevlett.129.267701 (2022).

[2] C.-X. Liu, S. Miles, A. Bordin, S. L. D. t. Haaf, A. M. Bozkurt, and M. Wimmer, Protocol for scaling up a sign-ordered kitaev chain without magnetic flux control (2024).

[3] M. Ezawa, Even-odd effect on robustness of majorana edge states in short kitaev chains, Physical Review B **109**, 10.1103/physrevb.109.l161404 (2024).

[4] V. Svensson and M. Leijnse, Quantum dot based kitaev chains: Majorana quality measures and scaling with increasing chain length, Physical Review B **110**, 10.1103/physrevb.110.155436 (2024).

[5] M. Luethi, H. F. Legg, D. Loss, and J. Klinovaja, The fate of poor man's majoranas in the long kitaev chain limit (2024).

[6] S. Miles, D. van Driel, M. Wimmer, and C.-X. Liu, Kitaev chain in an alternating quantum dot-andreev bound state array, Physical Review B **110**, 10.1103/physrevb.110.024520 (2024).

[7] A. Bordin, G. Wang, C.-X. Liu, S. L. ten Haaf, N. van Loo, G. P. Mazur, D. Xu, D. van Driel, F. Zatelli, S. Gazibegovic, G. Badawy, E. P. Bakkers, M. Wimmer, L. P. Kouwenhoven, and T. Dvir, Tunable crossed andreev reflection and elastic cotunneling in hybrid nanowires, Physical Review X **13**, 10.1103/physrevx.13.031031 (2023).
